# One Hole, Two Tubes, and a Tijuana Pathology Report: A Case Report of Cholecystoduodenal Fistula Mistaken for Gallbladder Cancer

**DOI:** 10.7759/cureus.6802

**Published:** 2020-01-28

**Authors:** Karim Jreije, Shawn Steen, Garrett Jones, Joseph A Eisner

**Affiliations:** 1 General Surgery, Community Memorial Hospital, Ventura, USA; 2 Surgery, Ventura County Medical Center, Ventura, USA

**Keywords:** cholecystoduodenal fistula, biliary fistula, gallbladder carcinoma

## Abstract

We report a case of a patient who presented with biliary colic while in Tijuana, Mexico. Laparoscopic cholecystectomy was attempted but abandoned and only a biopsy of the gallbladder was performed with pathologist reporting gallbladder adenocarcinoma. Upon return to the United States, extensive evaluation was undertaken including imaging, biopsy, and ultimately two separate exploratory surgeries revealing no neoplasm. Only at the second surgical exploration did we discover a benign cholecystoduodenal fistula successfully treated with completion fenestration cholecystectomy, pyloric exclusion, loop gastrojejunostomy, and duodenostomy tube through the gallbladder remnant into the fistula itself. This is a unique surgical treatment of a rare problem made even more confusing by an erroneous pathology report from another country.

## Introduction

It is estimated that 1-2% of all cholecystectomies performed for suspected benign disease will incidentally be found to have gallbladder cancer [1]. Furthermore, the presence of gallstones poses a known risk of gallbladder cancer, and 70-90% of all patients with gallbladder carcinoma also have gallstones. Despite these numbers, less than 0.5% of patients that have gallstones go on to develop gallbladder cancer [2].

Similarly biliary fistulas are quite rare, of which there are two types, internal and external and for which an estimated 2% will be from gallbladder cancer [3]. The external fistulas, which are abnormal connections between the gallbladder and subcutaneous tissues, are much more rare, and may result from suppurative cholecystitis or trauma [4]. While biliary fistulas are a rare phenomenon, there are reports of patients with multiple internal fistulae with different etiology [5]. Of the internal fistulas, which are abnormal connections between biliary tract and other organs, by far the majority (75%) are from connections between the gallbladder and duodenum, while the remainder are from connections between the gallbladder to the stomach, or large intestines and are likely to be the result of gastrointestinal inflammatory conditions [6,7]. Despite the infrequency with which these are encountered it is paramount to be aware of their existence, both in pre-operative workup and during management, as the mortality rate for cholecystoduodenal fistulas has been reported as high as 36.2% [8].

Similar to our experience, these fistulas, described by Courvoisier at the turn of the nineteenth century, have long posed unique challenges in diagnosis and treatment [9]. A high clinical suspicion for biliary fistula should be considered in the setting of abdominal pain, poor oral intake, in the presence of pneumobilia on imaging [10]. In many cases, the diagnosis may not be made prior to operative intervention, and patient could instead be diagnosed with peptic ulcer disease [11]. Aguilar-Espinosa et al. have proposed that a biliary fistula should be considered in elderly patients with a contracted gallbladder and numerous adhesions [12]. Of the recent publications on the topic of biliary fistula, computed tomography (CT) and MR cholangiography (MRCP) have been utilized to identify these abnormal connections [13]. Once identified, whether pre-operatively or intra-operatively, the recommended surgical plan is for cholecystectomy and fistulotomy with repair of the fistula [14].

## Case presentation

A 50-year-old Mexican American man was referred to surgical oncology at our stateside hospital for evaluation of gallbladder adenocarcinoma. He was diagnosed after he developed an acute attack of biliary colic while traveling in Tijuana, Mexico. At that time, a laparoscopic cholecystectomy turned partial cholecystectomy was reportedly aborted as intraoperative findings suggested locally advanced gallbladder cancer. The patient returned to the United States and presented to our hospital system five months after the initial biopsies in Mexico with recurrent right upper quadrant abdominal pain for which general surgery was consulted. He had no other significant past medical history, and family history was non-contributory.

**Figure 1:**
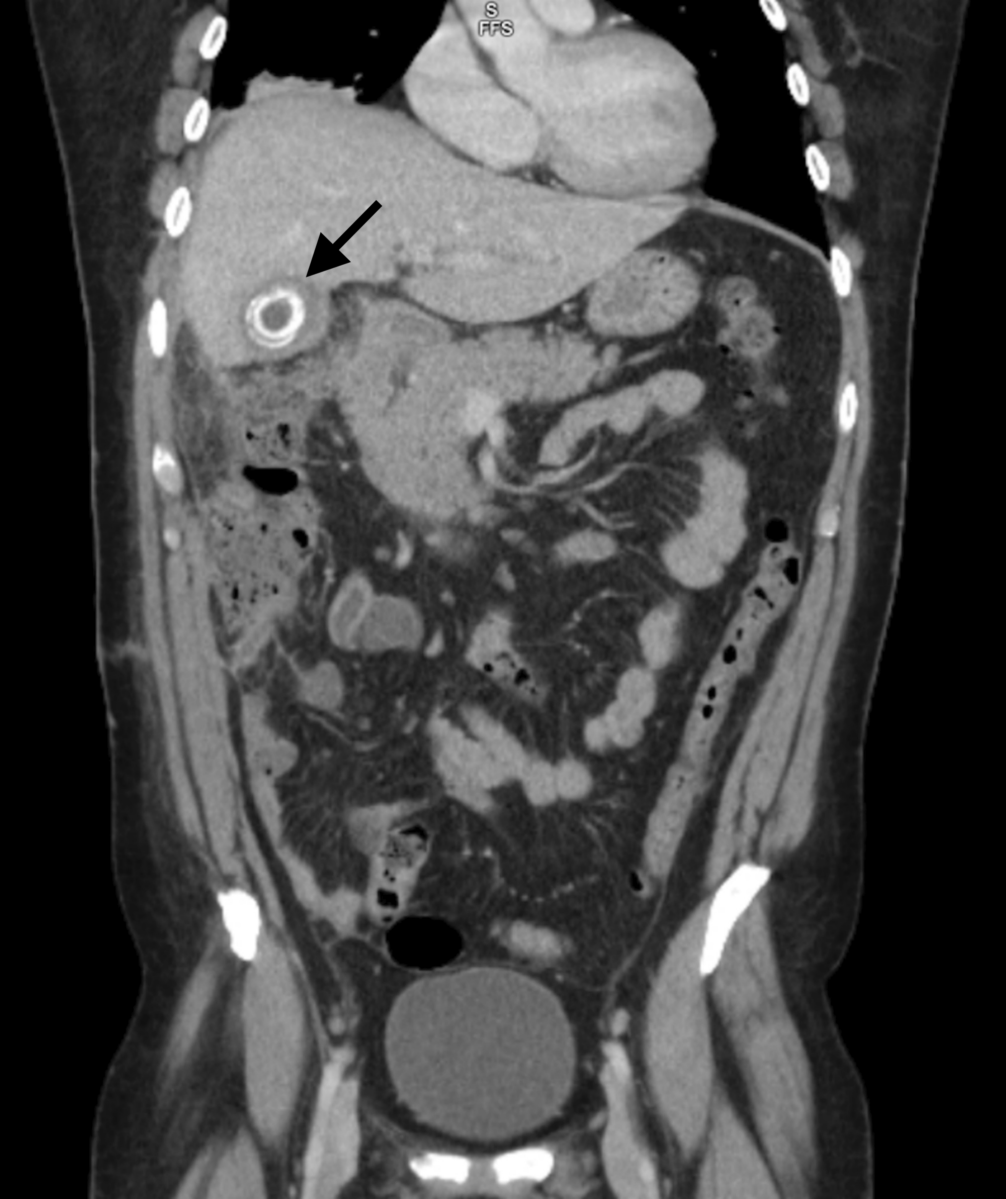
CT scan, coronal plane of calcifications within the gallbladder and surrounding inflammatory changes.

**Figure 2:**
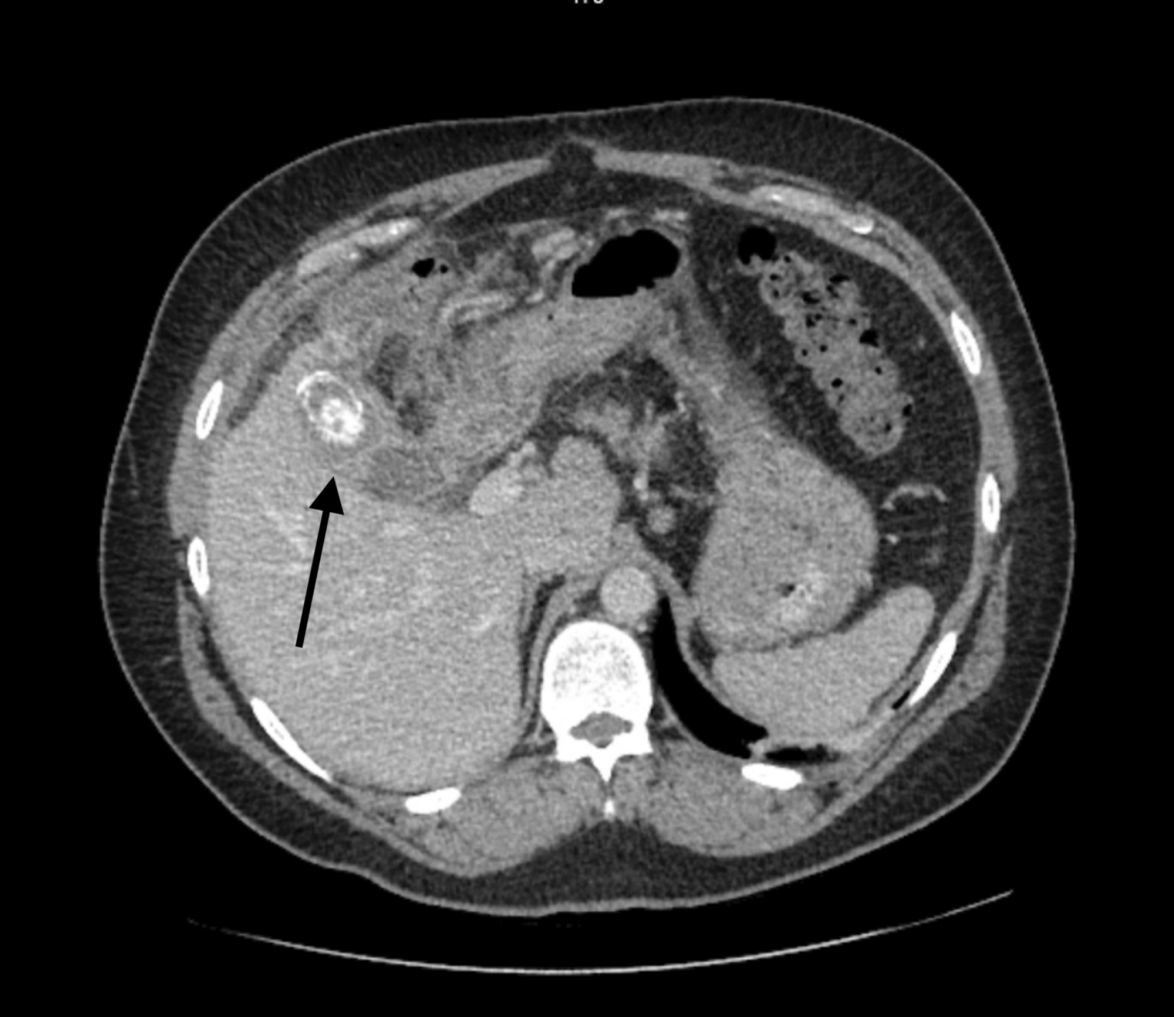
CT scan with significant inflammatory changes in the right upper quadrant, surrounding a gallstone; malignancy cannot be ruled out.

Examination revealed an obese, well-nourished man with normal vital signs and well-healing laparoscopic surgical incisions. He had a mild leukocytosis of 11,000 (normal < 10,000), alanine transaminase (ALT) 45, and alkaline phosphatase 182. CA19-9 levels were normal. A CT abdomen/pelvis (Figures 1, 2) revealed a large gallstone >3 cm and inflammation of the gallbladder fossa without any biliary obstruction or any hepatic or peritoneal metastatic disease.

Ultimately, he was discharged on ciprofloxacin and metronidazole and instructed to follow up with both medical and surgical oncology. We were unable to obtain the actual pathology slides from Mexico and our oncology team did not feel comfortable proceeding with treatment without confirmatory tissue diagnosis of malignancy.

He was subsequently scheduled for diagnostic laparoscopy which was more than one year after the initial attack in Mexico, but ultimately underwent exploratory laparotomy to obtain multiple liver, gallbladder, and omental biopsies of what appeared to be locally advanced unresectable gallbladder cancer, which was densely adherent to the duodenum, porta-hepatis, and hepatic flexure of the colon to the point where no discernible plane of dissection remained.

**Figure 3:**
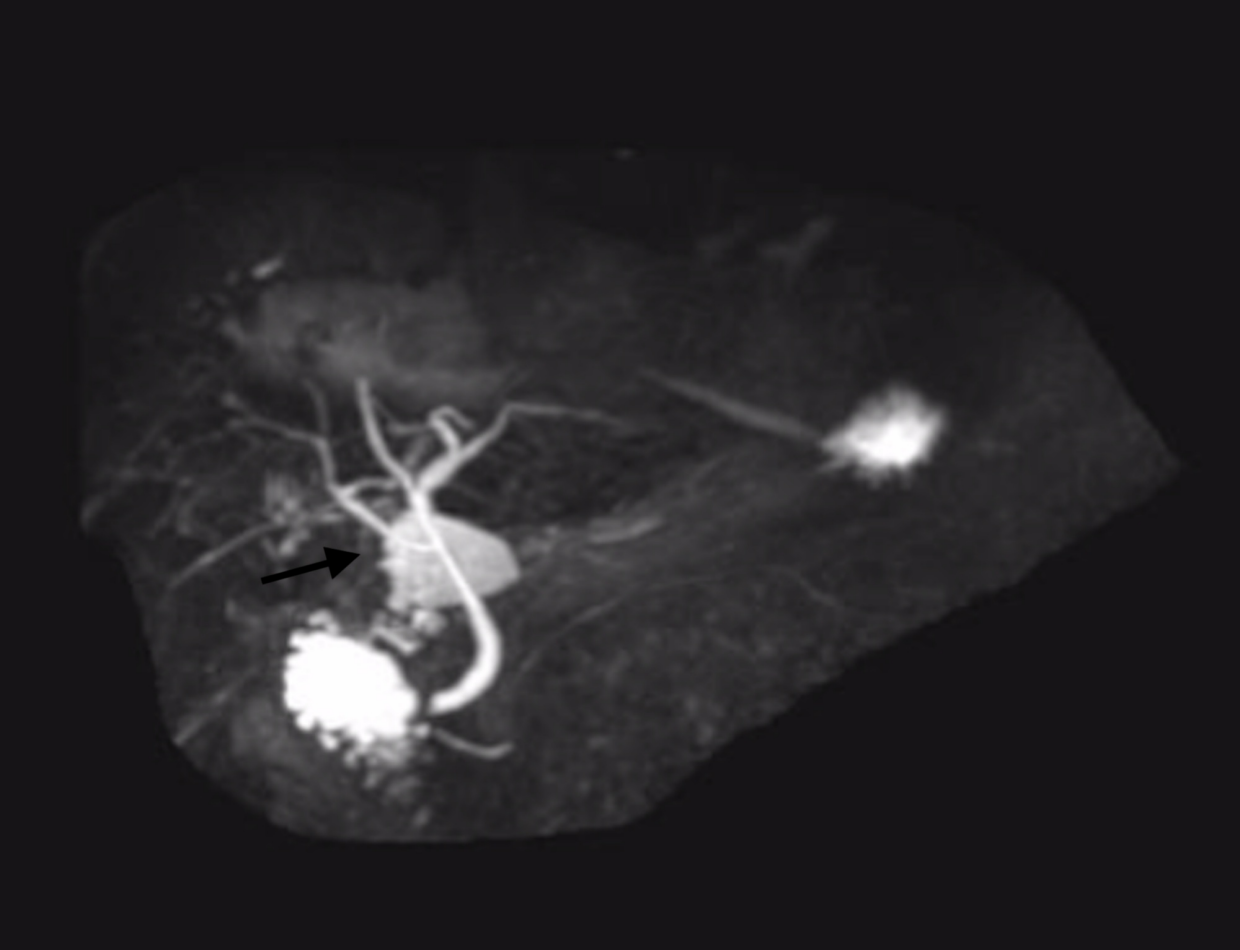
MR cholangiography (MRCP) image prior to surgery showing no bile duct obstruction. No evidence of cholecystoduodenal fistula reported in this study.

Postoperative pathology review after this initial surgical exploration in the U.S. revealed benign fat necrosis and chronic inflammation of all biopsied tissues. Positron emission tomography (PET) scan was then performed that demonstrated inflammation or tumor of the gallbladder without evidence of metastatic disease. Subsequent ultrasound-guided biopsy of the liver and gallbladder was also benign inflammatory tissue. MRCP demonstrated what appeared to be benign acute cholecystitis with a large 4 cm gallstone and uniform thickening of the gallbladder wall, proximate colon, and duodenum (Figure 3). The patient continued to experience biliary colic without weight loss or other symptoms. Given the time frame of nearly six months since the original pathology report of malignancy without progression of metastatic disease, weight loss, and other classic gallbladder cancer symptoms, malignancy seemed less likely.

**Figure 4:**
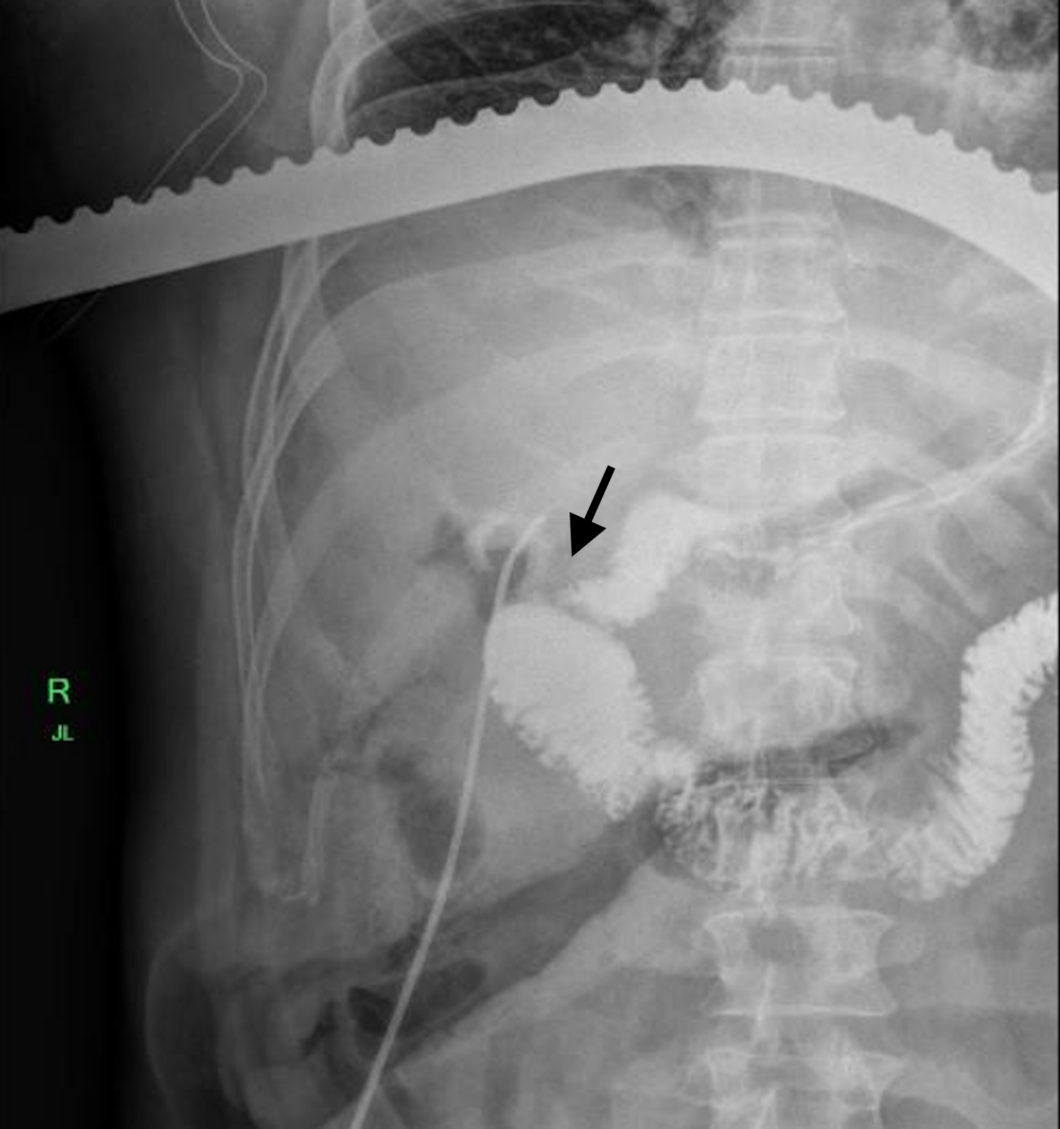
Intraoperative image showing dye injected through gallbladder lumen with clear fistulous connection to the duodenum.

After another three months to allow for diagnostic testing and for the inflammation to decrease, the patient was taken to the operating room for a second time and abdomen entered with a right subcostal incision. Intraoperative findings revealed many adhesions but no evidence of metastatic cancer. A dome down approach was used, and the top of the gallbladder was removed. A large 4 cm gallstone was removed and sent for pathologic evaluation. The top half of the gallbladder wall was removed in stepwise fashion and multiple frozen sections were sent. All tissue pathology was benign. Only the upper 2/3 of the gallbladder could be removed safely as it was densely adherent to the colon, duodenum, and porta-hepatis in the lower gallbladder. Digital palpation of the lower gallbladder lumen revealed what felt like a fistulous opening. A 16 Fr foley catheter was placed through the gallbladder, and dye was injected revealing contrast flowing directly into the duodenum, consistent with a cholecystoduodenal fistula of approximately 1 cm diameter, about 4 cm distal to the pylorus (Figure 4). No dye was observed within the biliary system. Given the obliteration of anatomic planes, no cystic duct dissection was performed. Intraoperative upper endoscopy confirmed the cholecystoduodenal fistula.

**Figure 5:**
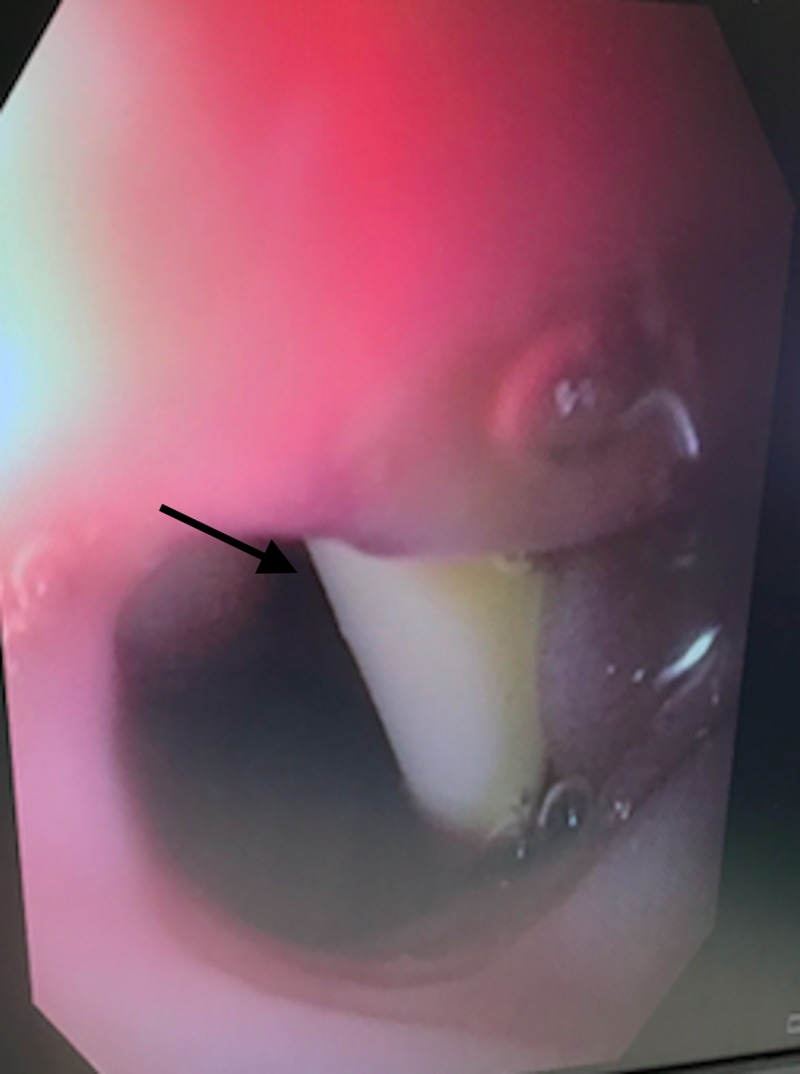
Intraoperative endoscopy showing the duodenostomy tube coming through the gallbladder lumen into the lumen of the duodenum.

Under direct visualization, the foley balloon was filled and confirmed visually to be through the remaining gallbladder lumen and also through the fistula and left in place to act as a duodenostomy tube (Figure 5). No evidence of a malignant mass was observed in the duodenum near the area of the fistula. An omental flap was placed directly into the gallbladder lumen and sutured in place, and a pyloric exclusion was completed with side to side gastrojejunostomy. A drain was placed directly over the fenestrated gallbladder. A nasogastric tube was left in the stomach for continued decompression postoperatively.

The patient’s immediate postoperative recovery was unremarkable. Diet was advanced to regular by the fourth post-operative day. Jackson-Pratt (JP) drainage remained serosanguineous, and he was discharged with all drains in place on postoperative day 7. Prior to discharge the final pathology report revealed chronic inflammation without evidence of malignancy.

He re-presented to the emergency department one week later with chief complaint of nausea and vomiting. A CT abdomen/pelvis was unremarkable. He was admitted with acute kidney injury and metabolic acidosis felt to be due to large volume of alkaline bile losses through the duodenostomy tube. He improved with rehydration and was discharged home on oral bicarbonate and fluid replacement.

By four weeks after surgery, his JP drain was removed and the duodenostomy tube was clamped and subsequently removed by six weeks after surgery. He had no further complaints, all right upper quadrant pain resolved, and he remains well with a follow-up of over one year.

## Discussion

The majority of biliary fistulae result from abnormal connections between the gallbladder and duodenum with the most common cause of cholecystoduodenal fistulas arising from direct erosion of a gallstone through the wall of the gallbladder [15]. It is estimated that approximately 90% are caused by gallstones [9]. The remaining 10% come from various other sources, such as duodenal ulcers or malignancy. With our patient, we found ourselves in a perplexing situation based on a foreign pathology report calling the severely inflamed gallbladder tissue a malignancy. As all surgeons know, it can be extremely difficult to differentiate malignancy from severe inflammation based on palpation and intraoperative tissue characteristics. Additionally, on pathologic examination of small volume of tissue, severe inflammation can have bizarre cells that can look like a malignancy. Despite liberal use of intraoperative pathologic evaluation during our initial surgical exploration, it was not determined that this was a truly malignant process and yet we were unaware of the benign cholecystoduodenal fistula and so did not initially pursue the surgical treatment for this problem. It took extensive testing and evaluation to convince ourselves that there was no malignancy and to feel confident proceeding with the more aggressive surgery that he finally received. We had considered endoscopic ultrasound as a diagnostic test but the gastroenterologist had declined the procedure after the MRI was read as benign cholecystitis without any mass concerning for malignant growth. The endoscopic ultrasound would have likely found the cholecystoduodenal fistula and would have made the pre-surgical planning different but may not have changed the final surgical outcome as the surgery we performed was felt to be the safest option for the problem. Without the erroneous initial pathology report of malignancy, the total treatment course would likely have been different.

## Conclusions

The diagnostic dilemmas arising from this case are instructive, and the successful surgical treatment of this cholecystoduodenal fistula with fenestrated cholecystectomy, duodenostomy tube drainage and pyloric exclusion is rarely described for this situation. Of the many things learned from this case, it elucidates that once a process is deemed malignant, the treatment paradigm shifts drastically and it can be difficult to redirect that course back to the treatment of a benign problem. We demonstrate here that the use of the duodenostomy tube to control the fistulous output along with pyloric exclusion worked well and is a safe surgical option. We present this case as an addition to the resources a surgeon could use to approach the unfortunate patient with a gallbladder fistula.
